# Neonatal sevoflurane exposure induces impulsive behavioral deficit through disrupting excitatory neurons in the medial prefrontal cortex in mice

**DOI:** 10.1038/s41398-020-00884-5

**Published:** 2020-06-20

**Authors:** Linghua Xie, Yue Liu, Yuhan Hu, Bei Wang, Zhirui Zhu, Yilei Jiang, Yaojun Suo, Miaofeng Hu, Jing Gao, Rahim Ullah, Zhiyong Hu

**Affiliations:** 1 Department of Anesthesiology, The First Affiliated Hospital, The Children’s Hospital, Zhejiang University School of Medicine, Hangzhou, China; 2 Department of Anesthesiology, The Children’s Hospital, Zhejiang University School of Medicine, Hangzhou, China; 3 Department of Cell Biology, Yale University, New Haven, CT USA; 4 Department of Neurobiology, The Innovation Center for Brain Science, Institute of Neuroscience, Zhejiang University School of Medicine, Hangzhou, China

**Keywords:** ADHD, Scientific community

## Abstract

Sevoflurane, in particular multiple exposures, has been reported to cause the abnormal neurological development including attention-deficit/hyperactivity disorder (ADHD). This study is to investigate ADHD-like impulsivity in adult mice after repeated sevoflurane exposures at the neonatal stage. Six-day-old pups were exposed to 60% oxygen in the presence or absence of 3% sevoflurane for 2 h and the treatment was administrated once daily for three consecutive days. To assess the impulsivity, the cliff avoidance reaction (CAR) was carried out at the 8th week. Our results showed that repeated sevoflurane treatment increased the number of jumps and shortened the jumping latency in the CAR test. The cortices were harvested for immunostaining to detect c-Fos and calmodulin-dependent protein kinase IIα (CaMKIIα) expression in the medial prefrontal cortex (mPFC). We found that mPFC neurons, especially excitatory neurons, were highly activated and related to impulsive behavior. The activation viruses (AAV-CaMKIIα-hM3Dq) were injected to evaluate the effects of specific activation of mPFC excitatory neurons on impulsive behavior in the presence of clozapine-N-oxide (CNO). Likewise, the inhibitory viruses (AAV-CaMKIIα-hM4Di) were injected in the sevoflurane group to explore whether the mPFC excitatory neuronal inhibition reduced the impulsivity. Our results revealed that chemogenetic activation of mPFC excitatory neurons induced impulsive behavior whereas inhibition of mPFC excitatory neurons partially rescued the deficit. These results indicate that repeated sevoflurane exposures at the critical time induce impulsive behavior accompanied with overactivation of mPFC excitatory neurons in adult stages. This work may further extend to understand the ADHD-like impulsive behavior of the anesthetic neurotoxicity.

## Introduction

Attention-deficit/hyperactivity disorder (ADHD) is a chronic neurodevelopmental disorder manifesting primarily as attention deficit, hyperactivity, and impulsivity^1^. ADHD affects 5–8% of school-age children, in which 50–80% cases persist till adulthood^2^. Adult ADHD has complex manifestations. Currently, the core symptom used in adult ADHD diagnosis is impulsivity, defined as poorly conceived control deficit^3,4^. Impulsivity increases the risk of pathological gambling, substance abuse, and relationship failure. As the prevalence rate of adult ADHD is soaring over the world, therefore, neurobiological and psychosocial factors related to the etiology and symptom control of ADHD have been studied in depth—exemplified by genes, neurotransmitters, neural pathways, drugs, and education.

Patients with ADHD show remarkable variation in complicating factors and neuropsychological weaknesses^5^. Previous studies suggested that the impaired development of dopaminergic neurons is related to ADHD development^6^. In addition to disorders in neurotransmitter system, other studies indicated that ADHD is caused by abnormal structural and functional synapses in mPFC^6,7^, which is responsible for behavioral inhibition and executive functions. Recently, clinical studies reported that repeated exposures to inhalational anesthetics in children is associated with the development of ADHD^6,8–10^. This finding was further supported by an animal study, which revealed that ketamine administration caused ADHD-like hyperactive behavior^11^. However, whether anesthetics have a long-term effect on ADHD development, particularly impulsive behavior, remains uncertain. Given the potential issue of public mental health and social stability, identifying the links between general anesthesia and impulse control disorder is now of paramount importance. Sevoflurane, the most commonly used pediatric anesthetic, has been shown to act as a neurotoxin affecting synaptogenesis and neuronal morphology in animal studies^12,13^. Therefore, we hypothesize that exposure of the immature brain to sevoflurane may induce ADHD-like behavior conditions in later adulthood.

In this study, we exposed neonatal mice to sevoflurane and found it induced ADHD-like impulsive behavior in the cliff avoidance reaction (CAR) test in adult age. We revealed that sevoflurane permanently affected the mPFC excitatory neurons, which were involved in impulsive behavior. We also demonstrated that chemogenetic inhibition of the mPFC excitatory neurons rescued sevoflurane-induced impulsive behavior. These results suggest that sevoflurane-induced impulsive behavior is another type of long-term outcome to anesthetic neurotoxicity. Furthermore, excitatory neuronal activation in the mPFC may serve as a pathological mechanism for anesthetics-induced ADHD in adults.

## Materials and methods

### Animals

All procedures were performed following the National Institutes of Health Guidelines for the Care and Use of Laboratory Animals and approved by the Animal Advisory Committee at Zhejiang University. C57BL/6J mice were group-housed in a temperature-controlled room at the Animal Facility of Zhejiang University under a 12 h/12 h light/dark cycle. Food and water were available ad libitum. Male and female mice were allowed to meet for one night and then the females were housed individually. Time mated birth was monitored and pups were collected for further experiments.

### Exposure to sevoflurane

Animal groups were determined in a pilot study and randomly assigned to the sevoflurane group and control group before the experiment. At postnatal day 6 (P6), pups were placed in a translucent plastic chamber (20 × 10 × 10 cm^3^) with a layer of soda-lime and wet cotton on the bottom floor. The temperature of the chamber floor was maintained at 32 °C using a heating pad under the chamber. The chamber was connected to a sevoflurane vaporizer (Vapor 2000; Medical Systems, Inc., Germany) and continuously flushed with 60% oxygen balanced nitrogen (FIO_2_ = 0.6) at 2 L/min for 2 h with (the sevoflurane group) or without (the control group) 3% sevoflurane. The treatment was administrated once per day for 3 consecutive days. Mice were allowed to grow up to 8 weeks. Female mice were excluded to avoid the possible confounding effects of the estrous cycle and only males were used for further experiments.

### Cliff avoidance reaction (CAR)

One cohort of mice (*n* = 24) was evaluated for the CAR test. CAR is a behavioral paradigm used to investigate the ADHD-like impulsive behavior in mature rodents^14–16^. It depends on the natural tendency of animals to avoid falling from a height, which is more than twice the length of the animal^17^. On account of a control deficit and risk-taking choice, the impulsive mice were more active in exploring the cliff, which led to a jump from the high platform at a higher frequency. According to a previous study^18^, a round platform (an inverted glass beaker with 11 cm diameter and 15 cm height) placed in the center of the open field apparatus (45 × 45 × 45 cm^3^) was used for the CAR test. The behavioral experiments were conducted in a quiet room illuminated by dim light between 9 and 12 am. Before the tests, the mice (*n* = 24) were handled in a friendly manner once/day for 3 consecutive days to decrease the stress-related neuronal activation. On the test day, mice were first allowed to habituate in the test room for more than 30 min. Each mouse was then gently placed on the platform and the duration that it remained on the platform (from the initial placement until falling onto the floor) was recorded. During the whole 15 min testing period, the mice were repeatedly placed on the platform once they fell off, and the cumulative cliff-falling events were documented. The latency is defined as the duration when mice stayed on the platform within the 15 min testing period. The apparatus was cleaned with 75% ethanol between tests to prevent olfactory influence.

### Elevated plus-maze (EPM) test

Another cohort of mice (*n* = 16) were evaluated for the EPM test. The EPM consists of two open arms and two closed arms (30 × 5 cm^2^) in an arrangement that the two arms of each type are opposite to each other. The maze was placed at the height of 65 cm above the ground. A video camera was fixed above the maze to record the movements of mice. Each mouse was placed in the center area facing the open arms and allowed to explore freely for 5 min. Time spent in the open arms, the number of entries into the open arms and freezing time were calculated using the video-based ANY-maze system (Stoelting, USA). The apparatus was cleaned with 75% ethanol between tests.

### Immunohistochemistry

90 min after completing the CAR test, 6 mice were sacrificed under terminated anesthesia the CAR test according to a random number table, and then perfused with 4% paraformaldehyde (PFA). Their brains were harvested for immunohistochemistry analysis. Coronal brain sections (40-μm thickness) were cut by a freezing microtome (CM30503, Leica Biosystems, Germany). After 2-h blocking in 5% normal donkey serum (NDS; 017-000-121, Jackson ImmunoResearch Laboratories, Inc. USA), 1% bovine serum albumin (BSA; A2153, Sigma-Aldrich, USA), and 0.3% Triton X-100 (Sigma-Aldrich, USA), the sections were incubated with the primary antibodies (mouse anti–c-Fos [1:1,000; Abcam, ab208942] and/or rabbit anti–CaMKIIα [1:1000; Abcam, ab52476]) overnight at 4 °C. Brain sections were then incubated with secondary antibodies (Alexa Fluor 488 anti-mouse IgG [1:1,000; A-21202, Thermo Fisher Scientific] or Alexa Fluor 546 anti-rabbit IgG [1:1,000; A-10040, Thermo Fisher Scientific]). The processed brain sections were then mounted onto glass slides with 60% glycerol in PBS. Images were captured using a Nikon A1 laser-scanning confocal microscope (Nikon, Japan). The number of immune-positive cells was counted and analyzed by ImageJ (NIH, USA). DAPI (4’,6-2- phenylindole; 1:3,000; C1002; Beyotime) was used simultaneously to identify nuclei.

### Surgery and chemogenetic manipulation

Under sodium pentobarbital anesthesia (50 mg/kg, intraperitoneal injection (i.p.)), other cohorts were mounted in a stereotaxic apparatus (RWD Life Science, Shenzhen, China). After adjusting the position of the skull horizontally under a stereomicroscope (RWD Life Science, Shenzhen, China)^19^, a small craniotomy was made with a thin drill over the medial prefrontal cortex (typical coordinate: 1.9 mm anterior to Bregma; 0.34 mm lateral to the midline). Adeno-associated virus (AAV) carrying fusion genes for hM3D (AAV- CaMKIIα-hM3D(Gq)-mCherry), hM4D (AAV- CaMKIIα-hM4D(Gi)-mCherry), or mcherry (AAV-CaMKIIα -mcherry) were injected using a 10 µL micro-syringe (#701, Hamilton, USA) at a rate of 60 nL/min by micro-syringe pump (kdScientific, USA). Over a 10-min period, 80 nL viruses were injected at a depth of 2.4 mm from the Bregma. Mice with viral injections in the incorrect area or expressions beyond the targeted area (mPFC) were excluded from the study. The hM3Dq was injected unilaterally for activation (*n* = 7) and hM4Di (*n* = 8) was bilaterally injected for inhibition. The control adenovirus (mCherry only) was also injected unilaterally or bilaterally for corresponding control experiments. The viruses hM3Dq (AAV_2/9_, 3.6 × 10^12^ genomic copies per ml), hM4Di (AAV_2/9_, 3.6 × 10^12^ genomic copies per mL), or mcherry (AAV_2/9_, 3.54 × 10^12^genomic copies per mL) were made by HanBio (Shanghai, China).

The mice injected with the above viruses were received a saline i.p. to test the baseline of the CAR deficit. After that, mice were given CNO (1 mg/kg i.p.; clozapine-N-oxide; HY17366, MedchemExpress, USA) for virus-infected neuron manipulation.

### Electrophysiological recordings of brain slices

After a 3–4-week viral incubation, coronal sections including the mPFC were cut at a thickness of 300 μm using a vibratome (VT 1200S, Leica, Germany) in ice-cold cutting solution (234 mM Sucrose, 5 mM KCl, 1.25 mM NaH_2_PO_4_, 5 mM MgSO_4_, 26 mM NaHCO_3_, 25 mM dextrose, 1 mM CaCl_2_, oxygenated with 95% O_2_/ 5% CO_2_) as described previously^19^. Next, slices were incubated in the aerated (95% O_2_/5% CO_2_) artificial cerebrospinal fluid (ACSF: 124 mM NaCl, 2 mM KCl, 1.25 mM KH_2_PO_4_, 2 mM MgSO_4_, 2 mM CaCl_2_, 26 mM NaHCO_3_, and 10 mM D-(C) glucose, pH 7.4.) to recover for 30 min at 32 °C, and subsequently incubated for 1 h at room temperature. Brain slices were then transferred to a recording chamber. To test function of expressed hM3Dq or hM4Di protein, whole-cell patch-clamp recordings were applied with glass pipettes backfilled with an artificial intracellular fluid (120 mM K-gluconate, 15 mM KCl, 10 mM HEPES, 4 mM Mg-ATP, 0.3 mM Tris-GTP, 0.5 mM EGTA, adjusted to pH 7.3 with KOH, 285–290 mOsm). Pipettes were connected to the headstage of a Heka EPC 10 amplifier (Heka Elektronik, USA). Cells expressing viral-encoded fluorescent markers (mCherry) were visualized under microscope with infrared differential interference contrast optics (BX51WI, Olympus, Japan). Current-clamp recording was used to measure evoked action potentials in both CNO activation and inhibition experiment. After applying currents in steps of 20 pA (from 0 to 360 pA) neurons were recovered for 5 min. The brain slices were then perfused with ACSF containing 5 µM CNO. The same current-clamp procedure was performed 10 min after CNO perfusion.

### Statistical analysis

The sample size was estimated by software PASS.11 (NCSS, USA) according to the preliminary experimental results. Experienced researchers conducted independently the different experiments and blinded data collectors analyzed these results. All data are shown as mean ± SEM unless otherwise specified. Before analysis, all data underwent the Kolmogorov–Smirnov normality test. Comparisons between two groups were made with unpaired or paired Student’s *t*-test if data are normally distributed; otherwise, Mann–Whitney *U* test or Wilcoxon signed-rank tests were used. Variance was found to be similar between the groups as tested using the Levene test of homogeneity of variances. Two-way ANOVA followed by Bonferroni’s post-hoc test was used for multiple comparisons, as appropriate. *p*-value less than 0.05 was considered to be statistically significant, defined as *p < 0.05; **p < 0.01; ***p < 0.001; and ****p < 0.0001. Calculation was performed in GraphPad Prism TM 8.0 (version 8.0; GraphPad Software Inc., USA) and SPSS 22.0 (IBM.Corp., USA).

## Result

### General data

During sevoflurane exposure, pups were in pink color and respiration was maintained well and there were no deaths throughout the whole process.

#### Postnatal sevoflurane exposure showed ADHD-like impulsive behavior and anxiety-free in adulthood

**Fig. 1:**
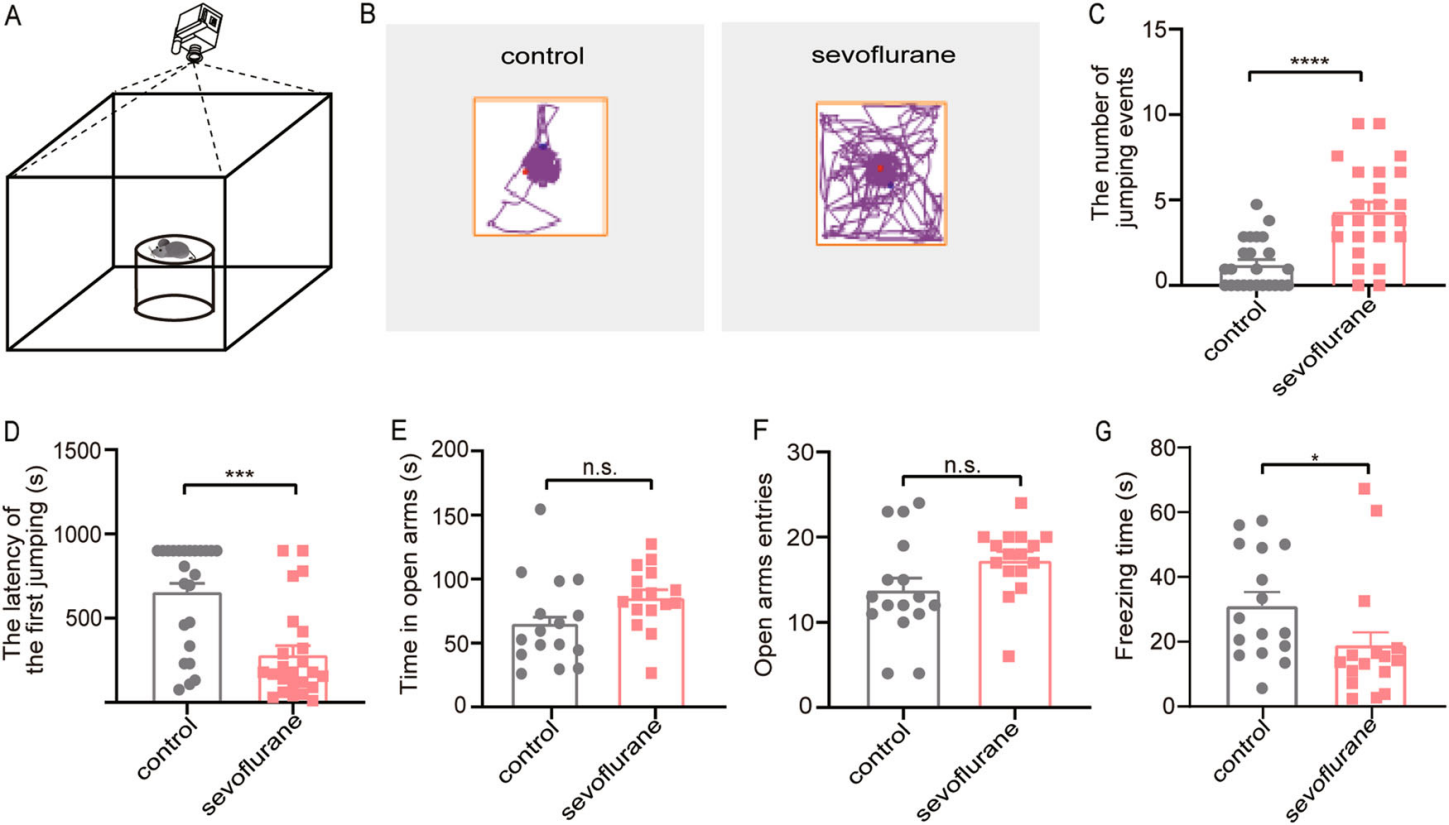
Postnatal sevoflurane exposure showed ADHD-like impulsive behavior and anxiety-free in adulthood. **a** Schematic diagram showing the cliff avoidance reaction (CAR) test paradigm. The mice were gently put on a platform during the CAR test. **b** The representative movement traces showing the locations of sevoflurane and control mice in the CAR test. **c**, **d** The number of cliff jumping events is higher and the latency of the first jump (the time from an initial placement on the platform until first falling) is significantly shorter in sevoflurane mice. (*n* = 24 for each group; Mann–Whitney *U* test). **e**–**g** The time spent in the open arms and the number of open arms entries of sevoflurane and control mice make no difference in an elevated plus-maze (EPM) test. Sevoflurane group shows less freezing time in the EPM test. (*n* = 16 per group; Unpaired Student’s *t* test for time spent in open arm; Mann–Whitney *U* test for open arm entries and freezing time.). Data = mean ± SEM; n.s. no significance, *p < 0.05, ***p < 0.001, ****p < 0.0001.

To investigate whether neonatal sevoflurane exposure induces voluntary risk-taking behavior in adulthood, the CAR test was performed on 8-week-old mice that have been treated either with sevoflurane or mock-control at the neonatal stage. An inverted glass beaker was placed in the center area of the open field apparatus, mice were carefully placed on the platform and the CAR test was initiated (Fig. 1a). After an adaptation period in the test room, each mouse was evaluated for 15 min and their movements were monitored by a video camera (Fig. 1b; Supplemental Video 1, 2). Sevoflurane-treated mice showed marked abnormalities with significantly increased jumping events (*p* < 0.0001, Fig. 1c) and decreased latency of falling from the platform compared with controls (*p* < 0.0001, Fig. 1d). A previous study showed that anxiety or fear itself could also result in impulsive-like behavior^20^. To exclude the contribution of anxiety and/or fear to impulsive-like behavior in our animal model, an EPM test was performed. The EPM test showed no significant difference in the time spent in open arms (*p* = 0.067, Fig. 1e) and entries to the open arms (*p* = 0.0594, Fig. 1f) between the groups. These data suggest that sevoflurane exposure has no impact on inducing anxiety. Moreover, the sevoflurane group exhibits significantly less freezing time (*p* = 0.0104, Fig. 1g). These results indicate that postnatal exposure to sevoflurane can induce ADHD-like impulsive behavior.

#### Enhanced mPFC neuronal activities are associated with the sevoflurane-induced impulsivity

**Fig. 2:**
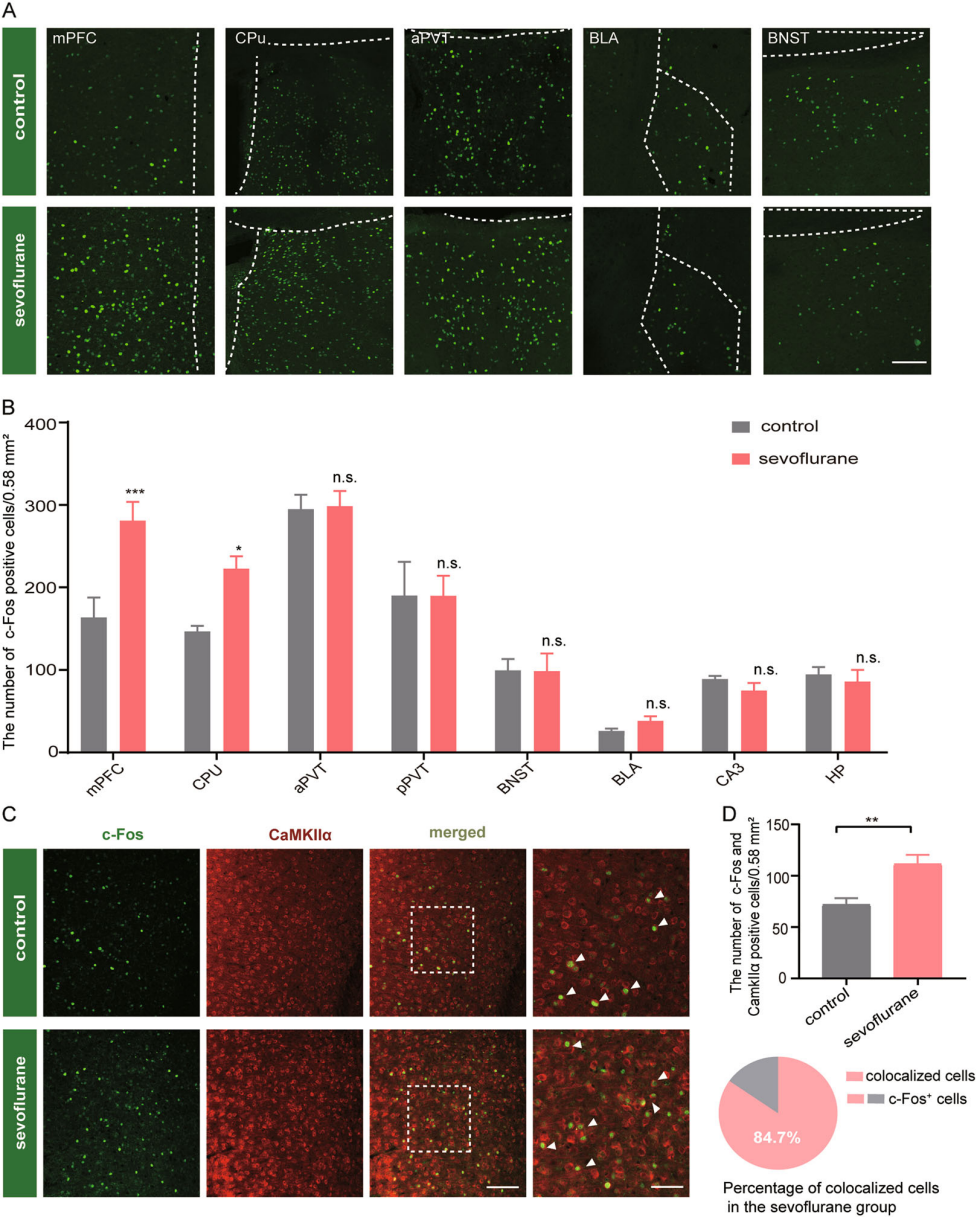
Enhanced mPFC neuronal activities are associated with the sevoflurane-induced impulsivity. **a** Representative images show the c-Fos-positive cells in mPFC, CPu, aPVT, BLA, BNST from control (top), and sevoflurane (bottom) mice. Scale bar, 100 mm. mPFC, medial prefrontal cortex; CPu, caudate putamen, striatum; aPVT anterior paraventricular thalamic nucleus; pPVT posterior paraventricular thalamic nucleus; BLA basolateral amygdaloid nucleus; BNST bed nucleus of the stria terminalis. (dotted line show the boundary of brain area). **b** Quantification of c-Fos positive cells in the mPFC, CPu, aPVT, pPVT, BNST, BLA, CA3, and HP, respectively. HP, hypothalamus area. The number of c-Fos expression in the mPFC is markedly higher for sevoflurane mice (*n* = 6, Two-way ANOVA, Bonferroni multiple comparison post hoc tests). **c** Representative micrograph showing the c-Fos^+^ neurons (green), CaMKIIα^+^ neurons (red) and colocalized cells. Scale bar (left) 100 μm; (right) 50 μm. (arrows = double-labeled cells). **d** Quantification of the number of c-Fos^+^ neurons that colocalized with CaMKIIα^+^ neurons (top). Percentage of CaMKIIα^+^ cells expressing c-Fos in the sevoflurane mice (bottom). (*n* = 6; Unpaired Student’s *t* test). Data = mean ± SEM; n.s. no significance, *p < 0.05, **p < 0.01, ***p < 0.001.

c-Fos has been reported as a reliable marker for neuronal activation and its expression is induced by various behavioral stimuli^21^. Therefore, we labeled brain sections with c-Fos to identify specific areas responsible for CAR behavior in our animal model. We counted the c-Fos expressing neurons in various brain regions, including mPFC, caudate putamen (CPu), anterior paraventricular thalamic nucleus (aPVT), basolateral amygdaloid nucleus (BLA) and bed nucleus of the stria terminalis (BNST) 90 min after the CAR test (Fig. 2a). Interestingly, sevoflurane-treated mice showed a notable increased expression of c-Fos in mPFC compared with control (F(7, 52) = 35.5, *p* = 0.0006, Fig. 2b). We found there were more CPu c-Fos positive cells in the sevoflurane-treated mice compared with control (*p* = 0.0382, Fig. 2b). No significant difference in c-Fos positive cells was identified between aPVT and posterior paraventricular thalamic nucleus (pPVT) between sevoflurane-treated mice and control (Fig. 2b). Furthermore, the c-Fos expression in anxiety- and fear-related brain regions, including BNST^22^ and BLA^23^ showed no significant difference between sevoflurane-treated mice and control, which is also consistent with the EPM results (Fig. 2b). It also showed that c-Fos expression in CA3 (an area of the hippocampus) and the hypothalamus (HP) has no significant difference between the treatment group and control (Fig. 2b).

Different neuronal subtypes in mPFC play various roles in encoding and regulating behaviors^24^. To examine what are the specific types of neurons that involve in impulsive behaviors, we performed double-immunofluorescent staining of CaMKIIα and c-Fos (Fig. 2c) for sevoflurane-treated mice and control. We found that sevoflurane-treated mice are possessing a significantly greater population of c-Fos positive cells expressing CaMKIIα (*p* = 0.0042, Fig. 2d). In the sevoflurane-treated group, 84.7 % of c-Fos positive cells were colocalized with the CaMKIIα^+^ neurons (Fig. 2d). Together these results suggest that excitatory neurons in mPFC were overactivated in sevoflurane-treated mice.

#### Chemogenetic activation of mPFC excitatory neurons induces ADHD-like impulsive behavior

**Fig. 3:**
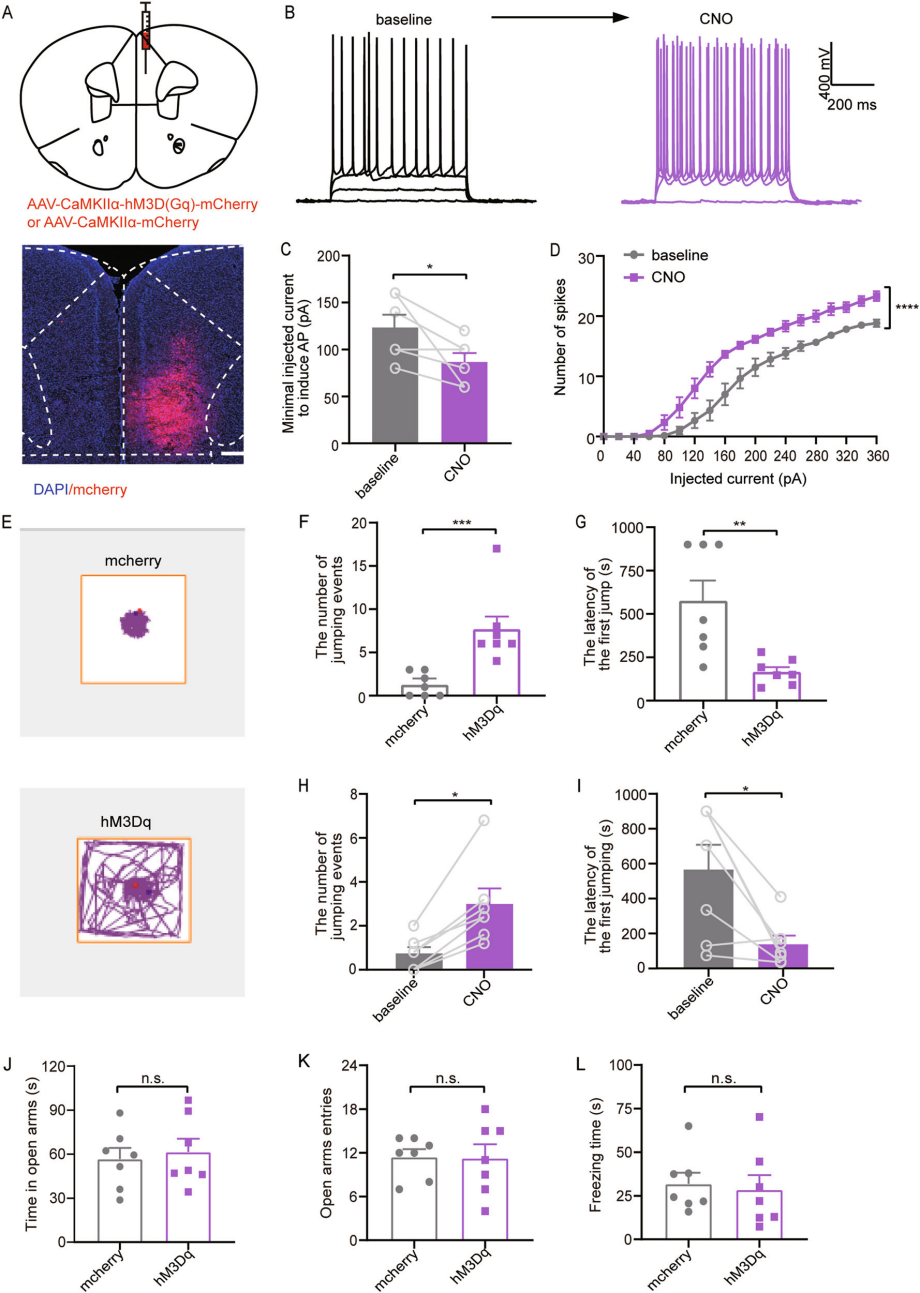
Chemogenetic activation of mPFC excitatory neurons induces ADHD-like impulsive behavior. **a** Schematic diagram (coronal section) showing the target of viral injection in the mPFC (top). Location of unilaterally viral hM3D (Gq) expression (red) in mPFC under control of the CaMKIIα promoter (bottom). **b** Current–voltage relationship of a representative mPFC neuron recorded before and during 5 μM CNO perfusion. Raw traces show individual voltage responses to a series of 600-ms current pulses from 0 to 360 pA with 20 pA steps. **c** CNO decreased the minimal injected current to induce action potential (AP). (*n* = 10, Paired Student’s *t* test). **d** The number of induced action potentials at different current steps. (*n* = 10, two-way ANOVA, Bonferroni multiple comparison post hoc tests.). **e** Representative movement traces showing the locations of mcherry-expressing (top) and hM3Dq-expressing in the air-exposure mice (bottom) in a cliff avoidance reaction test following CNO (1 mg/kg) administration. **f**, **g** Quantification of the number of cumulative cliff jumping events (left) and the latency (right) of the first jump from the cliff in the mcherry group and hM3Dq group (*n* = 7 per group; Mann–Whitney *U* test for the number; Unpaired Student’s t test for the latency). **h**, **i** Quantification of the number of cumulative cliff jumping events (left) and the latency (right) of the first jump from the cliff in the baseline group and hM3Dq group.(*n* = 7; Wilcoxon matched-pairs signed-rank test). **j**–**l** Quantification of the EPM results of the time spent in the open arms, open arms entries, freezing time of hM3Dq and mcherry group after chemogenetic activation of mPFC glutamatergic neurons. (*n* = 7; Unpaired Student’s *t* test). Data = mean ± SEM; n.s. no significance, *P < 0.05, **P < 0.01, ***P < 0.001, ****P < 0.0001.

It is known that mPFC is responsible for risk-decision making^4^. To test whether mPFC excitatory neural activities are involved in ADHD-like impulsive behavior, the DREADD (designer receptors exclusively activated by designer drugs)-based tools were used. We unilaterally injected excitatory virus (hM3Dq-mCherry) or non-functional control virus (mCherry) in mPFC (Fig. 3a). To ensure that the virus is effective, the whole-cell recording was performed in mPFC neurons. We confirmed that CNO administration activated hM3Dq-expressing neuronal activity in mPFC (Fig. 3b) by inducing a decreased spike threshold (*p* = 0.0478, Fig. 3c) and increased spike number under current step injections (Fig. 3d). Furthermore, we injected (i.p) CNO (1 mg/kg) into both mCherry-injected and hM3Dq-injected mice and traced the movements of hM3Dq-injected mice and control (Fig. 3e). We observed a significant increase in jumping events (*p* = 0.0006, Fig. 3f) and less latency (*p* = 0.0056, Fig. 3g) in the hM3Dq-injected group compared with the control group (mCherry-injected). Consistently, the activation of mPFC after CNO administration significantly increased the number of jumping events (*p* = 0.0156, Fig. 3h) and decreased the latency compared with the baseline level of CAR assessed by saline i.p. injection (*p* = 0.0156, Fig. 3i). Finally, we evaluated the anxiety level of mice using EPM test and found that time spent in the open arms (*p* = 0.6904, Fig. 3j) and the number of entries to the open arms (*p* = 0.9485, Fig. 3k) showed no significant difference between hM3Dq-injected mice and control. No significant difference was found in freezing time between the two groups (*p* = 0.7575, Fig. 3l). Together, our data indicate that chemogenetic activation of the mPFC neurons was sufficient to induce impulsive behavior deficit without affecting the freezing time or anxiety level.

#### Chemogenetic inhibition of neurons in the mPFC attenuates the sevoflurane-induced impulsive performance

**Fig. 4:**
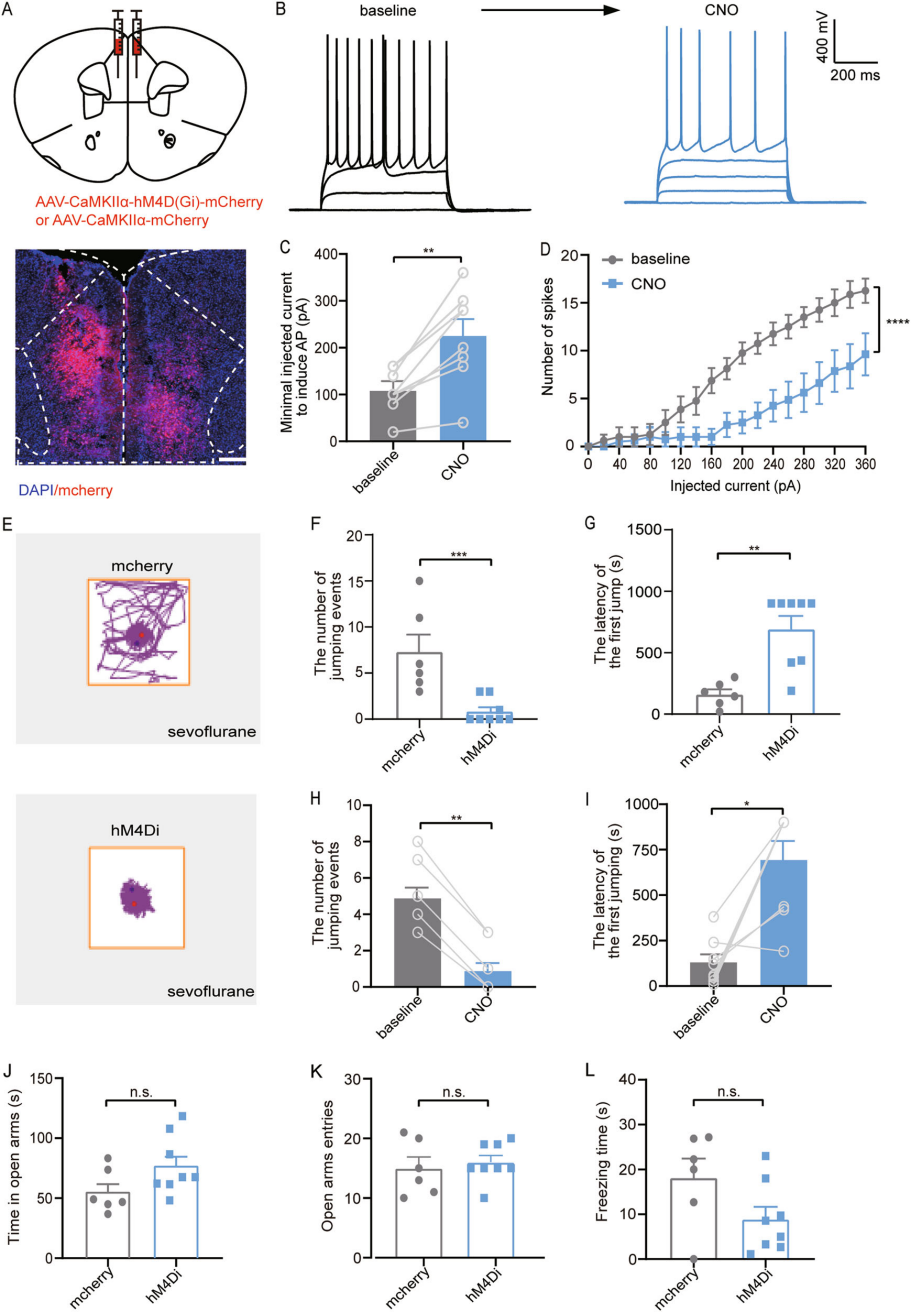
Chemogenetic inhibition of neurons in the mPFC attenuates the sevoflurane-induced impulsive performance. **a** Schematic diagram showing the target of viral injection (top). Location of the bilaterally viral hM4D (Gi) expression (red) in mPFC under control of the CaMKIIα promoter (bottom). **b** Current–voltage relationship of a representative mPFC neuron recorded before and during 5 μM CNO perfusion. Raw traces show individual voltage responses to a series of 600-ms current pulses from 0 to 360 pA with 20 pA steps. **c** The minimal injected current to induce action potential (AP) is increased by CNO. (*n* = 10; Paired Student’s *t* test). **d** The number of induced action potentials at different current steps. (*n* = 10; two-way ANOVA, Bonferroni multiple comparison post hoc tests). **e** Representative movement traces showing the locations of mcherry group (top) and hM4Di group (bottom) sevoflurane-exposure mice in a CAR test following CNO (1 mg/kg) administration. **f**, **g** Quantification of the number of cumulative jumping events (left) and the latency (right) of the mcherry group and hM4Di group. (*n* = 6, 8 for mcherry, hM4Di group respectively; Mann–Whitney *U* test). **h**, **i** Quantification of the number of cumulative jumping events (left) and the latency (right) of the baseline group and hM4Di group. (*n* = 8 for each group; Wilcoxon matched-pairs signed-rank test). **j**–**l** Quantification of the EPM results of the time spent in the open arms, open arms entries, freezing time of hM4Di and mcherry group after chemogenetic inhibition of mPFC glutamatergic neurons. (*n* = 6, 8 for mcherry, hM4Di group, respectively; Unpaired Student’s *t* test). Data = mean ± SEM; n.s. no significance, *P < 0.05, **P < 0.01, ***P < 0.001, ****P < 0.0001.

If the amplified activity of the mPFC excitatory neurons is responsible for impulsivity, then inhibition of these neurons may rescue the phenotype. To prove our hypothesis, we bilaterally injected hM4Di-mCherry and mCherry encoding virus into the mPFC in the sevoflurane-treated mice (Fig. 4a). The effectiveness of the viruses was also verified by electrophysiological recordings. CNO administration inhibited spiking in the hM4Di-expressing mPFC neurons (Fig. 4b), resulting in an increased spike threshold (*p* = 0.0013, Fig. 4c) and a reduction in spike number under the current step injection (Fig. 4d). Movements of mice were traced after CNO i.p. injection (Fig. 4e). We found that inhibition of the mPFC neurons dramatically reduced the jumping events (*p* = 0.0006, Fig. 4f) and increased the latency (*p* = 0.0023, Fig. 4g). Consistently, the CNO injection into the sevoflurane-treated mice showed fewer jumping events (*p* = 0.0078, Fig. 4h) and longer latency (*p* = 0.0156, Fig. 4i), compared with the baseline of the CAR assessed by saline i.p. injection. The administration of CNO has no effect on anxiety level (time in open arms, *p* = 0.095; open arms entries, *p* = 0.6412; Fig. 4j, k) or fear behavior (*p* = 0.0803, Fig. 4l). Our data suggest that inhibition of mPFC excitatory neurons ameliorate ADHD-like impulsive behavior for sevoflurane-treated mice.

## Discussion

These results demonstrated that repeated exposure to sevoflurane at the developmental stage causes ADHD-like impulsive behavior coupled with overactivated excitatory mPFC neurons later in adulthood. We also showed that selective activation of excitatory neurons potentially promoted impulsive behavior and chemogenetic inhibition of these neurons abolished the sevoflurane-induced impulsive behavior in adult mice. Our findings potentially provide insights for understanding the underlying mechanisms for anesthetics-related ADHD.

It has been reported that repeated, but not a single, anesthetic exposure during early postnatal development results in neurological impairment^25–27^, including neurocognitive impairments^28,29^. However, recent clinical studies indicated that children who received repeated anesthetic exposure had deficits in executive function and motor processing (ADHD)^30–32^. In this study, our finding suggests such ADHD-like impulsive behavior in adulthood may also due to exposure to sevoflurane during an early age (Fig. 1c, d). Some studies used 5-choice serial reaction time (5-CSRTT) to measure the impulsivity in rats, which required a long time training and learning^33^. Still, it is well established that neonatal exposure to sevoflurane is likely to impair animals’ memory or learning ability. We think the CAR test, which obtained impulsive-related measures from a free-exploration procedure without learning tasks, was more suitable for this type of study.

A previous study demonstrated that anxiety or fear itself can also result in impulsive-like behavior^20^. Therefore, to exclude fear- or anxiety-like behavior, we performed the elevated plus maze (EPM) test and found no anxiety alternation in both sevoflurane-treated group and viruses-injected mice. Intriguingly, the sevoflurane group showed less freezing time during the EPM test (Fig. 1g), which reflected sevoflurane-treated mice exhibited less fear. The reason may be that early exposure to sevoflurane has at least two behavioral consequences: increased impulsivity and fearlessness. Importantly, impulsivity and fear are clearly controlled by two separate circuits, as silencing the mPFC CaMKIIα^+^ neurons hardly reverses shortened freezing time shown in mice exposed to sevoflurane. This might also explain why the freezing time had no significant alternation (Figs. 3l and 4l) when the mPFC neuronal activities were manipulated.

The mPFC encodes and regulates the highest cognitive functions including impulse controlling^34,35^. The mPFC development is a continuous process and retains its neuroplasticity throughout the life^24^. Thus, sevoflurane could induce neurodevelopmental diseases by regulating synaptogenesis and synaptic development in the mPFC^36^, which suggests that mPFC neurons are vulnerable to sevoflurane-induced neurotoxicity. Indeed, our study found that neonatal exposure to sevoflurane-induced ADHD-like impulsive behavior at the adult stage through over-activating the mPFC excitatory neurons (Fig. 2b–d). Another clinical study suggested that glutamate level and the excitatory transmission have a modulatory role in ADHD adults^37^. We think that the dysfunctional glutamate system in the mPFC may be a pivotal contributor to adult ADHD development.

Based on our observation, the number of c-Fos^+^/CaMKIIα^+^ neurons in the mPFC is significantly increased in mice subjected to neonatal sevoflurane exposure. We believe that the effect is mainly due to the activation of the excitatory neurons. In general, anesthetics act on γ-aminobutyric acid (GABA) receptors to inhibit the neuronal activity and/or attenuating excitatory activity through the glutamate receptors. Prolonged inhibition of neuronal activity has been shown to induce homeostatic upregulation in neuronal excitability^38^. This intrinsic homeostatic plasticity triggered by relatively long-term sevoflurane exposure may be manifested during a critical developmental period. It will be interesting to examine whether the intrinsic biophysical properties of pyramidal neurons in the miniature excitatory postsynaptic current (mEPSC) in mice with early sevoflurane exposure are enhanced in future studies. Indeed, sevoflurane has been reported to enhance neuronal excitability through elevating corticosteroid levels^39^ and influencing the T-type Ca^2+^ channels^40^.

Both excitatory and inhibitory synaptic inputs have been shown to be regulated by sevoflurane during postnatal development. Lengthy sevoflurane exposure at P7 has been reported to compromise astrocyte development, which arrests excitatory synapse maturation and results in decreased mEPSC frequency in cortical pyramidal neurons^41^. Long-term reduction in excitatory inputs may cause homeostatic scaling of excitatory synapses a and indirectly enhance neuronal excitability. However, sevoflurane exposure at P16-17 only transiently increased mEPSC frequency and decreased miniature inhibitory postsynaptic current (mIPSC) frequency in mPFC pyramidal neurons in male mice^42^. Thus, it is essential to explore the detailed mechanism underlying early sevoflurane-induced alteration of synaptic integration in postsynaptic cells in future studies. Taken together, we believe that enhanced neuronal excitability in the mPFC in sevoflurane-treated animals may be due to a combination of direct changes in intrinsic properties and altered synaptic integrations of cortical neurons. Besides, the mPFC is composed primarily of glutamatergic neurons (80–90% of neurons) and inhibitory interneurons (10–20% of neurons)^43^. In our current study, about 15.3% of c-Fos positive cells in the mPFC were not excitatory neurons in the sevoflurane-treated mice during the impulsivity-related behavioral test (Fig. 2d), which indicates that few inhibitory neurons might be activated to balance the overactivation of excitatory neurons in the mPFC.

There are several limitations in this study. The mPFC has complex afferent and efferent projections to mediate the symptoms of psychiatric disorders^44^. Thus, how the downstream regions (e.g., thalamus, striatum, amygdala) of mPFC and their neural circuits are involved in processing behavioral changes in the sevoflurane-treated mice are unknown and warrant further study. Anesthesia and surgery are always going together. Our animals were surgical free and hence how surgery and associated trauma contribute to this behavioral change is also unknown and awaits further study.

## Conclusion

This study demonstrated that repeated sevoflurane exposure during the early development period induces ADHD-like impulsive behavior in later adulthood. This disorder is related to the overactivation of mPFC neurons and can be attenuated by the inhibition of mPFC excitatory neurons. These results may suggest that sevoflurane-induced impulsive behavior is another long-term outcome caused by the anesthetic neurotoxicity and the excitatory neuronal activation in the mPFC may serve as a pathological mechanism for anesthetics-induced ADHD in adults.

## Supplementary information

Supplemental Video 1

Supplemental Video 2
